# The relationship between quadriceps fat pad syndrome and patellofemoral morphology: a case–control study

**DOI:** 10.1186/s10195-021-00580-0

**Published:** 2021-04-28

**Authors:** Yavuz Yuksel, Tarkan Ergun, Ebru Torun, Melih Unal, Lena Sonnow, Ozkan Kose

**Affiliations:** 1 Department of Radiology, Faculty of Medicine, Alaaddin Keykubat University, Alanya, Turkey; 2 Department of Orthopedics and Traumatology, Yatagan State Hospital, Mugla, Turkey; 3 Department of Diagnostic and Interventional Radiology, Hannover Medical School, Hannover, Germany; 4 Department of Orthopedics and Traumatology, Antalya Training and Research Hospital, Kazım Karabekir Cd., Soguksu, 70100 Muratpaşa, Turkey

**Keywords:** Knee impingement syndromes, Quadriceps fat pad

## Abstract

**Background:**

The purpose of this prospective case–control study is to investigate the relationship between quadriceps fat pad syndrome (QFPS) and patellofemoral morphology.

**Materials and methods:**

Twenty-two patients with QFPS and 22 age- and gender-matched healthy volunteers were included. The diagnosis of QFPS was supported both clinically and radiologically. On magnetic resonance imaging (MRI), patellofemoral morphology was evaluated with 13 radiological measurements including trochlear sulcus angle, trochlear sulcus depth, trochlear facet asymmetry, trochlear condyle asymmetry, lateral trochlear inclination angle, patellar translation, tibial tubercle–trochlear groove (TT–TG) distance, Insall–Salvati ratio, patellotrochlear index, patellar tilt, the ratio between lateral and medial facet lengths, interfacet angle, and quadriceps tendon thickness. The mean of measurements was compared between groups using the Mann–Whitney *U* test.

**Results:**

There were 22 patients (12 male, 10 female) with mean age of 30.81 ± 1.41 (range 19–38) years in group I and 22 patients (12 male, 10 female) with mean age of 31.13 ± 1.31 (range 19–39) years in group II. The mean age and the gender distribution were statistically similar between groups (*p* = 0.845, *p* = 1, respectively). All measured values except for patellar tilt (*p* = 0.038) and TT–TG distance (*p* = 0.004) were similar (*p* > 0.05 for the other variables). However, all of the measured variables were within the normal range.

**Conclusions:**

QFPS may not be associated with anatomical variations of the patellofemoral joint. Further studies are required to understand the etiology and risk factors.

**Level of evidence:**

Level III, prospective case–control study

## Introduction

**Fig. 1:**
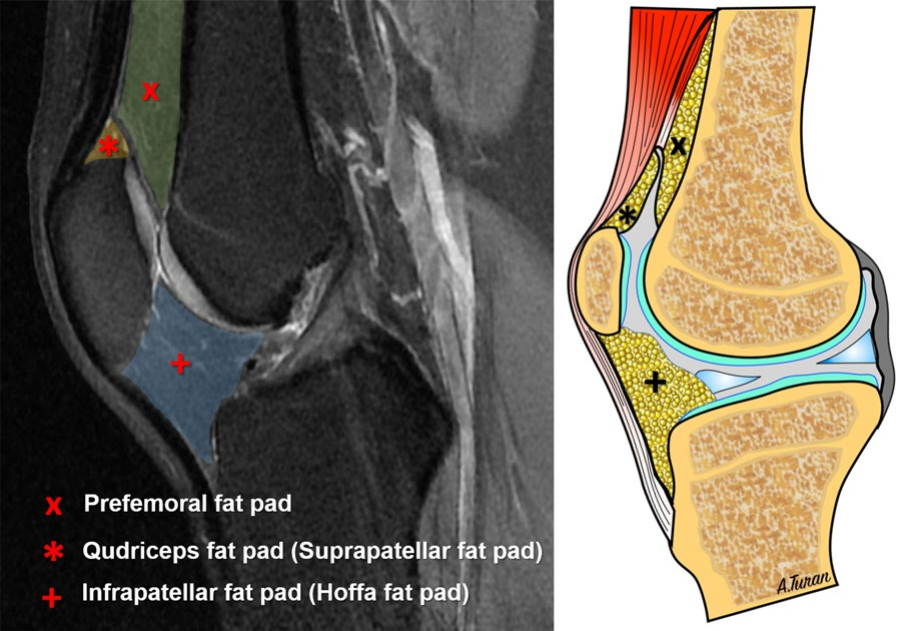
Sagittal fat-saturated, proton-density-weighted MRI image and corresponding illustration showing the location of the fat pads on the anterior aspect of the knee joint

The intraarticular fat pad is a mass of intracapsular but extrasynovial adipose tissue that occupies the potential spaces within the knee joint. They act as a protective cushion between the quadriceps and patellar tendon, patella, and distal femur, prevent friction, and improve patellofemoral engagement during deep knee flexion and extension [1–3]. Furthermore, it is proposed that they also provide proprioceptive sensation [4, 5]. There are three distinct fat pads located around the extensor mechanism of the knee joint: the infrapatellar fat pad, also called Hoffa fat pad, the pre-femoral fat pad, and the quadriceps fat pad [1] (Fig. 1). The quadriceps fat pad (QFP), also called the suprapatellar fat pad, is located between the quadriceps tendon and the suprapatellar recess of the knee joint. It is the smallest fat pad and triangular-shaped with average size of 8 ± 2 mm in men and 7 ± 2 mm in women [3].

**Fig. 2:**
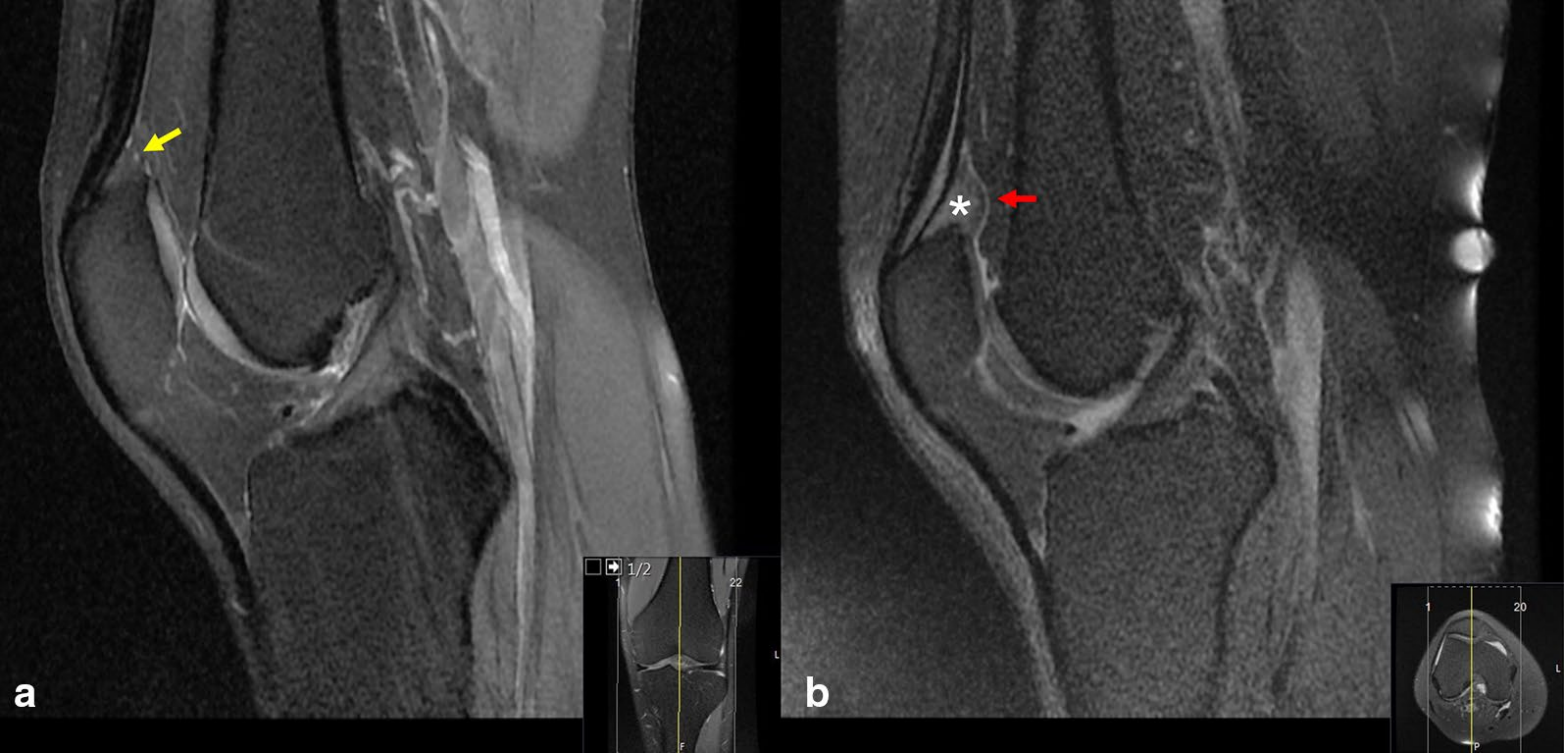
**a** Appearance of normal quadriceps fat pad (yellow arrow). **b** A patient with QPFIS. The sagittal fat-saturated, proton-density-weighted MRI demonstrates typical convex appearance (red arrow) and increased signal and thickening within quadriceps fat pad (white star)

Quadriceps fat pad syndrome (QFPS) is a rare cause of anterior knee pain, being characterized clinically by tenderness over the superior pole of the patella and pain on deep knee flexion [2, 5, 6]. Magnetic resonance imaging (MRI) shows hypertrophy with a typical convex appearance and discrete edema of the quadriceps fat pad characterized by high signal intensity on T2-weighted sagittal images (Fig. 2). In the absence of clinical findings, a diagnosis of QFPS cannot be made based solely on MRI findings because quadriceps fat pad edema (QFPE) may be present in the absence of anterior knee pain [2, 7]. In other words, QFPS is a clinical entity; however, QFPE is an imaging finding and may or may not be related to anterior knee pain [5].

The etiology of QFPS is not fully understood yet. Previously, a few studies have attempted to explain the presence of edema with accompanying patellofemoral (PF) abnormalities [2, 7–9]. Most of these studies reported a mass effect of the QFP, being an incidental finding on knee MRI in retrospective study designs. An association with anterior knee pain was assumed; however, there was often a lack of a valid clinical diagnosis of QPFS, and control groups were selected from patients with other knee disorders. Thus, the objective of this study is to select patients with a clinical diagnosis of QFPS and test for the relation to PF morphological characteristics compared with a group of healthy subjects in a prospective case–control study design.

## Patients and methods

### Patients and study design

This study comprised two groups of patients. The first group consisted of consecutive patients who were admitted to the orthopedic outpatient clinic between January 2016 and January 2019 with the complaint of anterior knee pain and were considered to have fat pad impingement syndrome following clinical evaluation performed by an orthopedic surgeon. During physical examination, tenderness over the suprapatellar region and increased pain in the same region with deep knee flexion were used as clinical criteria [6, 10]. The same orthopedic surgeon examined all patients. Two typical imaging findings were used for the radiological diagnosis of QFPS on MRI: (1) presence of QFPE characterized by high signal intensity in the QFP that was 20% higher than the surrounding normal fat pad signal, and (2) presence of QFP hypertrophy characterized by convexity of the posterior aspect of the QFP and/or bowing of the posterior margin of the quadriceps tendon due to expansion of the fat pad. The signal intensity of QFP and the normal fat pad (using the Hoffa fat pad as a reference) was measured on sagittal fat-saturated, proton-density-weighted MRI images with standard-sized regions of interest. The contrast between normal and abnormal fat pad was calculated as a percentage.

Thus, the diagnosis of QFPS was supported both clinically and radiologically in all patients. Patients with QFPE on MRI without confirmatory clinical symptoms were excluded. In addition, patients who underwent surgical intervention of the knee or who had history of fracture around the knee joint, markedly distorted knee anatomy due to congenital or acquired deformities, patellofemoral cartilage lesions (grade 2B or higher according to modified Noyes cartilage lesion classification), or incomplete or inadequate clinical and radiological data were excluded from group I [11].

The second group was composed of healthy volunteers who were matched for age and gender to the first group and who had no history of knee problems and whose clinical examination findings were completely normal. One volunteer with an asymptomatic cartilage lesion of the patella, which was incidentally detected, was excluded from the control group.

During the time under study, 22 (12 female, 10 male) patients with mean age of 30.81 ± 1.41 (range 19–38) years met the inclusion and exclusion criteria. Consequently, a sex- and age-matched control group composed of 22 volunteers (12 female, 10 male) with mean age of 31.13 ± 1.31 (range 19–39) years was prospectively formed. This study was carried out following the ethical standards laid down in the 1964 Declaration of Helsinki and its later amendments, and the institutional review board approved the study protocol (IRB approval date/issue: 10354421-2019/1-17.01.2019). Informed consent was obtained from all patients.

### MRI protocol

The MRI examination was carried out using a 16-channel knee coil with a 1.5-T MRI device (Symphony, Siemens, Erlangen, Germany). The imaging protocol included sagittal fast spin-echo, T1-weighted [repetition time (TR)/echo time (TE) 600/10, matrix 256 × 192, field of view (FOV) 16 × 16, slice thickness 3 mm], sagittal fat-saturated, proton-density-weighted (TR/TE 4000/30, matrix 256 × 192, FOV 16 × 16, slice thickness 3 mm), coronal fat-saturated, proton-density-weighted (TR/TE 2500/40, matrix 256 × 192, FOV 16 × 16, slice thickness 3 mm), and axial fat-saturated, proton-density-weighted (TR/TE 2900/40, matrix 256 × 192, FOV 16 × 16, slice thickness 4 mm).

### Observers and reliability analysis

All anatomic measurements were performed on digital MRI images stored in picture archiving and communication systems (PACS) by two independent observers using Sectra IDS7 software (version 18.2, Sectra AB, Sweden). Both observers were radiologists with more than 10 years of experience in musculoskeletal radiology (senior and second authors). Interobserver reliability was calculated using the interclass correlation coefficient (ICC) and 95% confidence interval. ICCs of 0.81–1.00, 0.61–0.80, 0.41–0.60, 0.21–0.40, and 0.00–0.20 were interpreted as excellent, good, moderate, fair, and poor, respectively [10]. All measurements showed excellent agreement with ICC ranging between 0.860 and 0.960. The measurements recorded by the senior author were used for the final analysis.

### Measurements on MRI

**Fig. 3:**
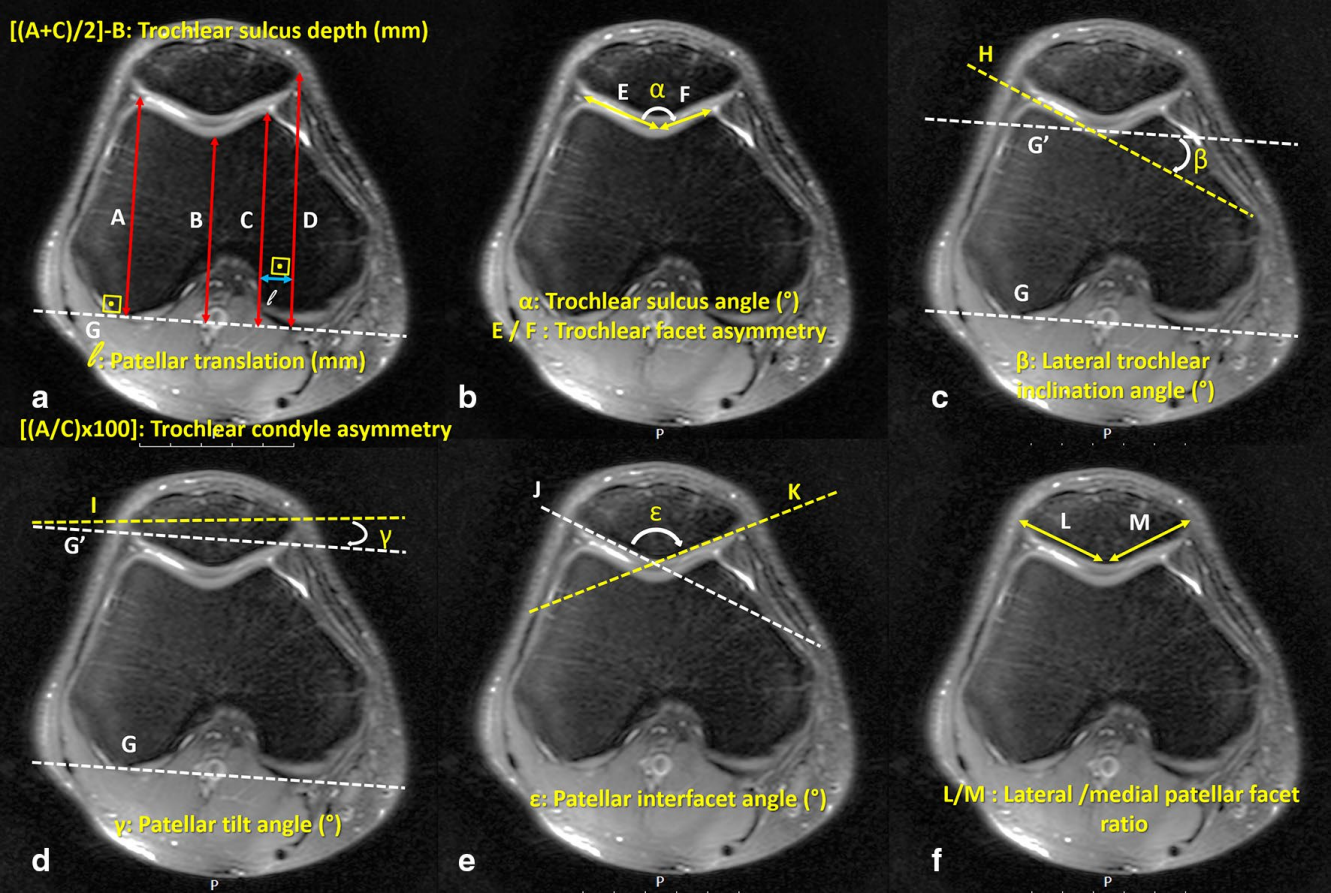
**a** Measurement of trochlear sulcus depth, patellar translation, and trochlear condyle asymmetry. First, posterior condylar axis was drawn (line G). Perpendicular lines from the highest points of the medial (line A) and lateral (line C) facets, and the deepest point (line B) of the trochlear groove were drawn. Finally, the perpendicular distance from the posterior condylar axis to the medial corner of the patella (line D) was drawn. Trochlear sulcus depth was calculated as [(*A* + *C*)/2] − *B* and recorded in millimeters. Patellar translation was measured as the perpendicular distance between line C and line D and recorded in millimeters. Trochlear condyle asymmetry was calculated as [(*A*/*C*) × 100%] and recorded as a percentage. **b** Measurement of trochlear sulcus angle and trochlear facet asymmetry. Two parallel lines, one to the lateral facet of the trochlea (line E) and the second one to the medial facet of the trochlea (line F), were drawn. The angle (*α*) between these lines was measured as the trochlear sulcus angle. The ratio of the distance line E to line F was calculated as the trochlear facet asymmetry. **c** Measurement of lateral trochlear inclination angle (*β*) between the lateral trochlear slope (line H) and posterior condylar axis (line G). **d** Patellar tilt was defined as the angle (*γ*) formed between the transverse axis of patella (line I) and posterior condylar condyle line (line G). **e** Patellar interfacet angle was defined as the angle between the lateral patellar facet (line K) and medial patellar facet (line J). **f** The ratio of the lateral (line M) to the medial (line L) patellar facets was calculated. All measurement were performed on axial fat-saturated, proton-density-weighted MRI

**Fig. 4:**
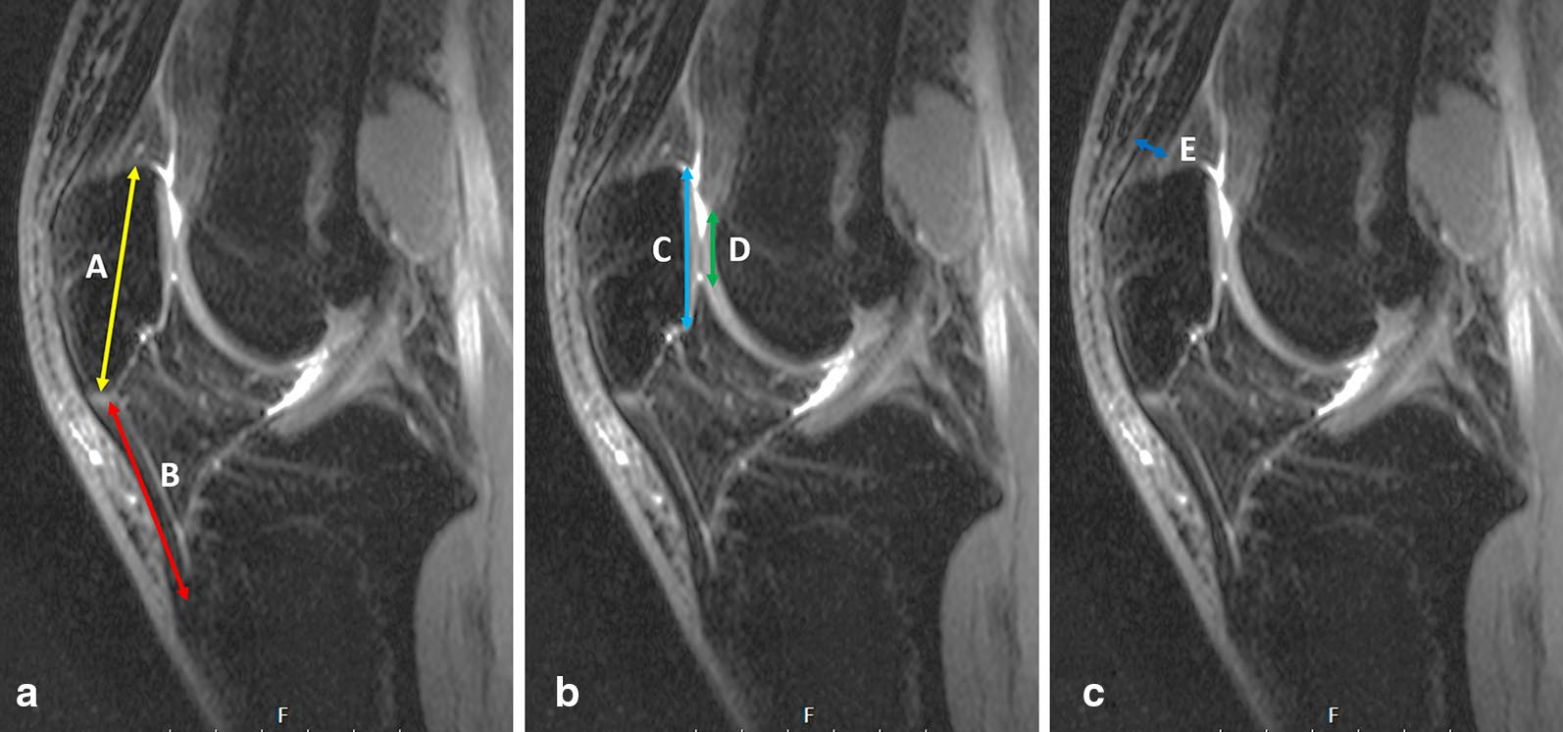
**a** The ratio of patellar tendon length (Line B) to longest diagonal length of patella (line A) was calculated as the Insall–Salvati ratio. **b** The ratio of length of articular surface of patella (line C) to length of opposing femoral articular surface (line D) was calculated as patellotrochlear index. **c** The distance between the most anterior and most posterior border of the quadriceps tendon just over the patellar attachment was measured and recorded as quadriceps tendon thickness. All measurements were performed on sagittal fat-saturated, proton-density-weighted MRI

**Fig. 5:**
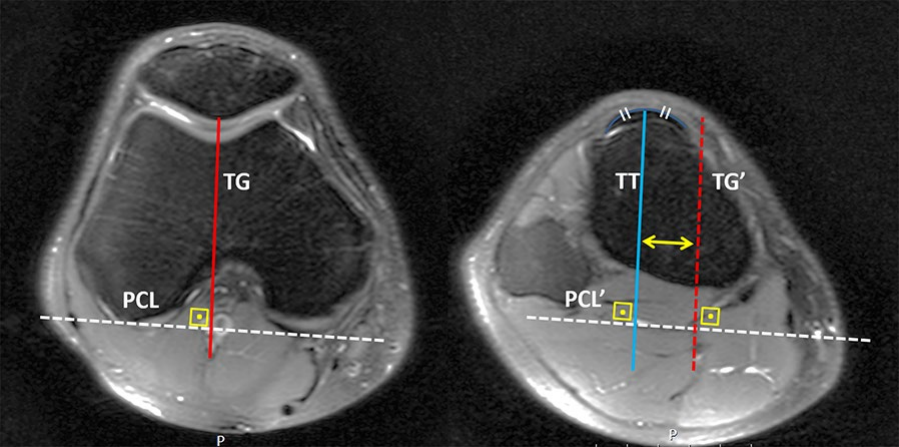
Measurement of tibial tubercle (TT) to trochlear grove (TG) was performed on two overlapping axial images. PCL, posterior condylar line; TG, perpendicular line from deepest point of trochlear groove to posterior condylar line; TG, perpendicular line from the tibial tubercle to posterior condylar line. TT–TG distance is the distance between red and blue lines

To assess the trochlear and patellar morphology and the patellofemoral joint anatomy, (1) trochlear sulcus depth, (2) patellar translation, (3) trochlear condyle asymmetry (Fig. 3a), (4) trochlear sulcus angle, (5) trochlear facet asymmetry (Fig. 3b), (6) lateral trochlear inclination angle (Fig. 3c), (7) patellar tilt (Fig. 3d), (8) patellar interfacet angle (Fig. 3e), (9) lateral to medial patellar facet ratio (Fig. 3f), (10) Insall–Salvati ratio (Fig. 4a), (11) patellotrochlear index (Fig. 4b), (12) quadriceps tendon thickness (Fig. 4c), and (13) tibial tubercle–trochlear groove distance (Fig. 5) measurements were performed according to previous descriptions [3, 12–19]. Moreover, all patients were classified as normal or abnormal in accordance with the previously accepted normal range. Detailed descriptions of the measurement techniques are explained in figure legends.

### Statistical analysis

Statistical analysis was performed using SPSS Statistics Base v.23 for Windows by the Department of Statistics. Continuous variables are presented as mean ± standard deviation, median, and range. The Kolmogorov–Smirnov test was used to determine whether the data were distributed normally. Comparative analysis of two independent groups was performed using the Mann–Whitney *U* test, Student’s *t*-test, and chi-square test. *p* < 0.05 was accepted as statistically significant.

## Results

There were 22 patients (12 male, 10 female) with mean age of 30.81 ± 1.41 (range 19–38) years in group I and 22 volunteers (12 male, 10 female) with mean age of 31.13 ± 1.31 (range 19–39) years in group II. The mean age and the gender distribution were statistically similar between groups (*p* = 0.845, *p* = 1, respectively). All measured values except for patellar tilt (*p* = 0.038) and TT–TG distance (*p* = 0.004) were similar (*p* > 0.05 for the other variables) (Table 1). However, all of the measured variables were within the normal range (patellar tilt < 20°, TT–TG distance < 15 mm).

**Table 1:** Comparative data between normal individuals and QFPS group

Variable	Control group	QFPI group	*p*-value*
Trochlear sulcus angle (° ± SD)	124.1 ± 1.1	123.2 ± 1.1	0.560
Trochlear depth (mm ± SD)	8.24 ± 0.29	8.47 ± 0.23	0.670
Trochlear facet asymmetry (ratio ± SD)	0.72 ± 0.025	0.71 ± 0.018	0.836
Trochlear condyle asymmetry (ratio ± SD)	1.02 ± 0.005	1.02 ± 0.006	0.902
Lateral trochlear inclination angle (° ± SD)	26.96 ± 1.01	28.77 ± 0.87	0.224
Patellar translation (mm ± SD)	1.32 ± 0.44	1.78 ± 0.58	0.238
Patellar tilt (° ± SD)	5.37 ± 0.75	3.25 ± 0.66	*0.038*
Lateral-to-medial facet length ratio (ratio ± SD)	1.16 ± 0.019	1.13 ± 0.024	0.201
Interfacet angle (° ± SD)	132.14 ± 1.29	130.66 ± 1.39	0.481
Insall–Salvati ratio (ratio ± SD)	1.07 ± 0.02	1.04 ± 0.03	0.173
Patellotrochlear index (ratio ± SD)	0.19 ± 0.015	0.18 ± 0.02	0.990
TT–TG distance (mm ± SD)	10.92 ± 0.57	8.01 ± 0.67	*0.004*
Quadriceps tendon thickness (mm ± SD)	6.32 ± 0.203	6.44 ± 0.191	0.742

*Mann–Whitney *U* test. Italic *p* values are significant

A post hoc power analysis was conducted using G*Power software (version 3.1.9.6). It has been reported that the most valuable radiological parameters to detect the presence of patellar maltracking reliably were TT–TG distance and patellar tilt [20]. Thus, the mean values of these variables were used for the calculation. With a sample size of 22 in each group and a two-tailed 5% significance level, power of 100% was reached for both variables.

## Discussion

The aim of the current study is to investigate the relationship between certain anatomical characteristics of the patellofemoral joint and QFPS. This small fat pad (FP) is a component of the extensor mechanism of the knee joint and has various functions during knee motion; thus, any alteration of the PF anatomy may cause excessive pressure that might lead to inflammation and, consequently, hypertrophy as a compensatory tissue reaction. In this prospective case–control study of patients with clinically diagnosed QFPS and radiologically confirmed mass effect of the QFP, most of the measured anatomical parameters of the PF joint did not differ significantly compared with a group of healthy subjects. Consistent with previous, mostly retrospective studies, it remains difficult to find a certain association between QFPS and measurable morphological changes, e.g., trochlear dysplasia or malalignment of the patella. In our study, we measured the TT–TG distance to be about 2–3 mm lower in the QFPS group than in the control group; however, we do not value this result as evidence for a changed morphology as the difference is low and there can be different results according to patient positioning during MRI examination. It is common to measure lower TT–TG in slight flexion compared with full extension due to the final external rotation of the tibia. Presumably, patients with knee pain are less willing to fully extend their knee during the examination, which must be considered during examination and measurement. The same conclusion can be drawn regarding the patellar tilt angle, which was about 2° higher in the control group, indicating stronger external rotation of the patella compared with the femur.

QFPE and the mass effect were first described by Roth et al. in 2004 [2]. They proposed that this imaging finding may not be incidental and might be related to anterior knee pain, anatomic variations of the extensor mechanism, and other coexisting knee abnormalities. In their study, MRIs of 92 knees were retrospectively reviewed, and 11 patients (12%) with quadriceps fat pad mass effect were identified. However, they could not show any association between anterior knee pain and the measured anatomic characteristics, or other accompanying lesions, between the patients with or without QFPE. They proposed three possible theories to explain the presence of quadriceps fat pad edema. The first theory was based on the impingement and the abnormal biomechanics of the extensor mechanism. Secondly, they thought that this might be an overuse injury and caused by chronic repetitive hyperflexion of the knee joint. Finally, it might be induced by other unrelated pathologies within the knee joint as a reactive inflammation. However, none of these theories could be proved or rejected with their data and study design.

In another retrospective study, Shabshin et al. reviewed 770 knee MRIs and detected quadriceps fat pad edema and mass effect in 32 knees of 29 patients (4.2%) [7]. However, less than one-third of patients (27.6%) had anterior knee pain. They investigated the relationship between QFPE and PF cartilage lesions, quadriceps tendon abnormalities, and patellar tendinosis but could not show any significant correlation. However, one of the patients in their cohort underwent biopsy and subsequent resection of the quadriceps fat pad. Pathological examination revealed vasculitis with obliteration of small vessels, and the patient’s symptoms were completely relieved. Based on their findings, they proposed that QPFE might be a new clinical entity analogous to Hoffa’s disease. During the same time interval, Sirvanci and Ganiyusufoglu reported that they had treated four patients who had QPFE with steroid injection [10]. A biopsy was also taken from one patient, and myxoid degeneration and inflammation were seen. Similarly, Van Le et al. reported one more patient who improved with US-guided steroid injection [21]. All these findings support that QFPE is not an incidental MRI finding alone but rather a distinct clinical entity characterized by inflammation and pain.

To understand the etiology of this new clinical entity, Tsavalas et al. retrospectively examined a large series of knee MRI (*n* = 879) and identified 110 (12.8%) patients with QFPE and mass effect [8]. Anterior knee pain was present in only six of these patients, and again they could not detect any significant difference in patellofemoral alignment and cartilage lesions between the case and control groups. As mentioned above, QFPE and clinical impingement syndrome are two different entities. While one of them describes a clinical condition, the other is just a radiological finding. The presence of radiological findings does not always mean that impingement findings or related anterior knee pain will occur. These studies are retrospective screening studies, and both case and control groups were collected incidentally from patients who already have knee problems. Therefore, neither the case nor control group was genuinely representative.

There is only one study in the relevant literature that contradicts our findings. Recently, Can et al. studied the relationship between the severity of QPFE (quadriceps fat pad signal intensity, dimensions, and posterior indentation) and the PF alignment and several anatomic characteristics of the PF joint [9]. There were no significant differences between severity groups with morphological measurements, but at least one pathological finding was present in 55 out of 61 patients (90.2%). They concluded that QFPE is related to pathologies related to the extensor mechanism and recommended that patellofemoral pathologies should be considered when QFPE is detected in knee MRI. However, a single measurement parameter associated with QFPS could not be elucidated.

Apart from these studies, two further studies have examined the relationship between QFPE and knee osteoarthritis. Wang et al. reported that QFPE and mass effect were associated with knee pain, radiographic findings of osteoarthritis (OA), and bone marrow lesions [22]. They proposed that QFPE might be a component of the knee OA disease, considering that OA is not confined to the cartilage and subchondral bone but involves all tissues surrounding the knee joint. In contrast, these findings were refuted with the study conducted by Fontenella et al. The morphological characteristics (volume and dimensions) of quadriceps fat pad were compared between normal, moderate OA, and end-stage OA groups [23]. No difference was determined among groups, suggesting that it is not clearly involved in OA. Because of these contradictory findings, it is not clear that the quadriceps fat pad is influenced by the pathogenesis of OA. Similarly, none of the patients in our case group had signs of knee OA. However, it should be noted that these patients were young subjects under 40 years of age.

There is only one clinical study dealing with the treatment of QFPS [6]. In this study, patients were allocated to steroid injection and physical therapy groups. In both groups, significant improvement was seen at 6 months follow-up. More importantly, QFPE regressed in control MRI studies. The findings of this study indirectly support our findings. Resolution of inflammation (both clinically and radiologically) without any intervention on the preexisting anatomical characteristics shows us that QFPS could be a transient inflammatory disease.

There are a number of strengths and limitations of this research. An important limitation of the study is the low case number due to the fact that clinically and radiologically confirmed QFPS is a rather rare diagnosis and patient data are limited. Moreover, there is no specific physical examination finding for the clinical diagnosis of QFPS; thus, the clinical diagnosis of patients might miss other causes of anterior knee pain, despite extensive efforts for exclusion. MRI is an expensive imaging modality, which limited the number of controls. Secondly, the radiologists could not be blinded to the presence or absence of edema during the measurements, which might be a potential bias. Finally, the lack of T2-weighted pulse sequence in the MRI protocol is another limitation. Besides these limitations, the current study has strengths, too. The control group was selected from healthy volunteers, and the diagnosis of QFPS was supported by clinical and MRI findings.

## Conclusions

This prospective case–control study could not show any relationship between patellofemoral anatomy and the QPFS. Further studies on the etiology of this rare syndrome should be performed and should focus on other factors.

## Data Availability

The data are available upon request to the corresponding author.

## Abbreviations

ICC: Interclass correlation coefficient
IRB: Institutional review board
MRI: Magnetic resonance imaging
OA: Osteoarthritis
PACS: Picture archiving and communication systems
PF: Patellofemoral
QFP: Quadriceps fat pad
QFPE: Quadriceps fat pad edema
QFPS: Quadriceps fat pad syndrome
US: Ultrasound
TT–TG: Tibial tubercle–trochlear groove
